# Correction: Use of a humanoid robot for auditory psychophysical testing

**DOI:** 10.1371/journal.pone.0336343

**Published:** 2025-11-05

**Authors:** Luke Meyer, Laura Rachman, Gloria Araiza-Illan, Etienne Gaudrain, Deniz Başkent

In Fig 2, the incorrect values have been provided for the Bayes factor. The values 0.001 and 0.003 should read “0.01” and “0.03” respectively. Please see the correct Fig 2 here.

**Fig 2:**
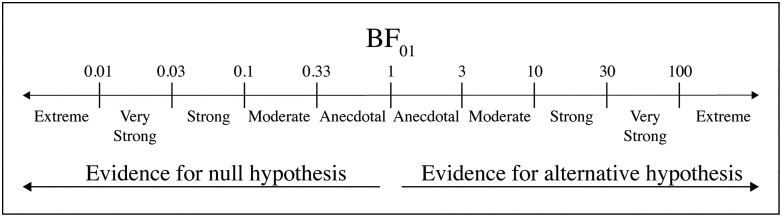
Classification scheme for the Bayes factor (BF01) by JASP.
